# Accessibility of Percutaneous Biopsy in Retrocolic-Placed Pancreatic Grafts With a Duodeno-Duodenostomy

**DOI:** 10.3389/ti.2024.12682

**Published:** 2024-08-06

**Authors:** Clara Bassaganyas, Anna Darnell, Alexandre Soler-Perromat, Gerard Rafart, Pedro Ventura-Aguiar, Miriam Cuatrecasas, Joana Ferrer-Fàbrega, Carmen Ayuso, Ángeles García-Criado

**Affiliations:** ^1^ Radiology Department, Centre Diagnòstic per la Imatge (CDI), Hospital Clinic de Barcelona, Barcelona, Spain; ^2^ Universitat de Barcelona, Barcelona, Spain; ^3^ Nephrology and Kidney Transplant Department, Institut Clínic de Nefrologia i Urologia (ICNU), Hospital Clinic de Barcelona, Barcelona, Spain; ^4^ Pathology Department, Centre de Diagnòstic Biomèdic (CDB), Hospital Clinic de Barcelona, Barcelona, Spain; ^5^ Hepatobiliopancreatic Surgery and Liver & Pancreas Transplant Department, Institut Clínic de Malalties Digestives i Metabòliques (ICMDM), Hospital Clinic de Barcelona, Barcelona, Spain; ^6^ Agust Pi i Sunyer Biomedical Research Institute (IDIBAPS), Barcelona, Spain

**Keywords:** pancreas transplantation, biopsy, ultrasound-guided biopsy, percutaneous, duodeno-duodenostomy

## Abstract

Duodeno-duodenostomy (DD) has been proposed as a more physiological alternative to conventional duodeno-jejunostomy (DJ) for pancreas transplantation. Accessibility of percutaneous biopsies in these grafts has not yet been assessed. We conducted a retrospective study including all pancreatic percutaneous graft biopsies requested between November 2009 and July 2021. Whenever possible, biopsies were performed under ultrasound (US) guidance or computed tomography (CT) guidance when the US approach failed. Patients were classified into two groups according to surgical technique (DJ and DD). Accessibility, success for histological diagnosis and complications were compared. Biopsy was performed in 93/136 (68.4%) patients in the DJ group and 116/132 (87.9%) of the DD group (*p* = 0.0001). The graft was not accessible for biopsy mainly due to intestinal loop interposition (n = 29 DJ, n = 10 DD). Adequate sample for histological diagnosis was obtained in 86/93 (92.5%) of the DJ group and 102/116 (87.9%) of the DD group (*p* = 0.2777). One minor complication was noted in the DD group. The retrocolic position of the DD pancreatic graft does not limit access to percutaneous biopsy. This is a safe technique with a high histological diagnostic success rate.

## Graphical Abstract

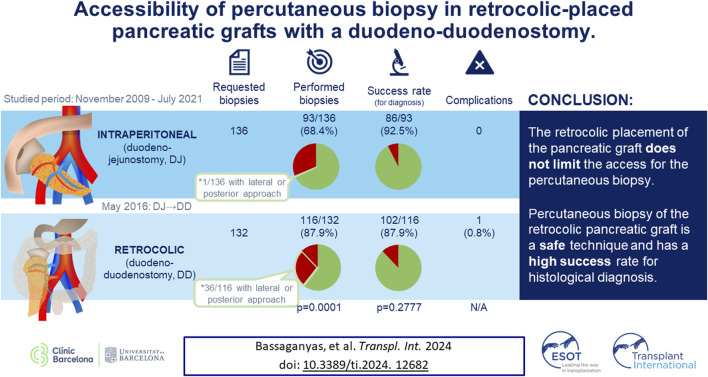

## Introduction

During the last decades, several surgical and therapeutic advances in pancreas transplantation have improved both patient and graft survival [1, 2]. However, graft rejection is one of the main causes of graft failure (25% of the grafts) [3] and remains a diagnostic challenge. Clinical manifestations and laboratory markers are non-specific [4, 5]. Although imaging studies are key to rule out many other causes of graft dysfunction (graft pancreatitis, vascular events, relapse of type 2 diabetes mellitus …) [6–8], they do not provide specific findings for diagnosing graft rejection. Pancreatic graft biopsy is therefore the gold standard for diagnosing graft rejection. Histological evaluation provides additional information, distinguishing cellular from antibody-mediated rejection, grading its severity and excluding other causes of dysfunction.

There are different techniques for obtaining pancreatic graft samples. Laparoscopic access offers a good success rate for histological diagnosis (around 95%) [9] but it is more expensive, less available, and has been reported to have a 2.5% conversion to laparotomy [9, 10]. The endoscopic approach is less invasive but it is a complex technique only performed in some centers [11–13], with low accessibility rates [13] and a low success rate for histopathological diagnosis (50%) [11, 12]. Besides, it mainly uses sampling of the graft duodenal mucosa (instead of sampling the pancreatic graft), whose utility in diagnosing graft rejection is still under debate [14–16]. Percutaneous access is the most widely used technique because it has been demonstrated to be safe and effective for the classical intraperitoneal positioning of the graft, whether guided by ultrasound (US) [17, 18] or computed tomography (CT) [19]. In addition, it is a simple and cheap technique that does not require sedation or an operating room, thereby contributing to decreased costs and occurrence of comorbidities.

**FIGURE 1:**
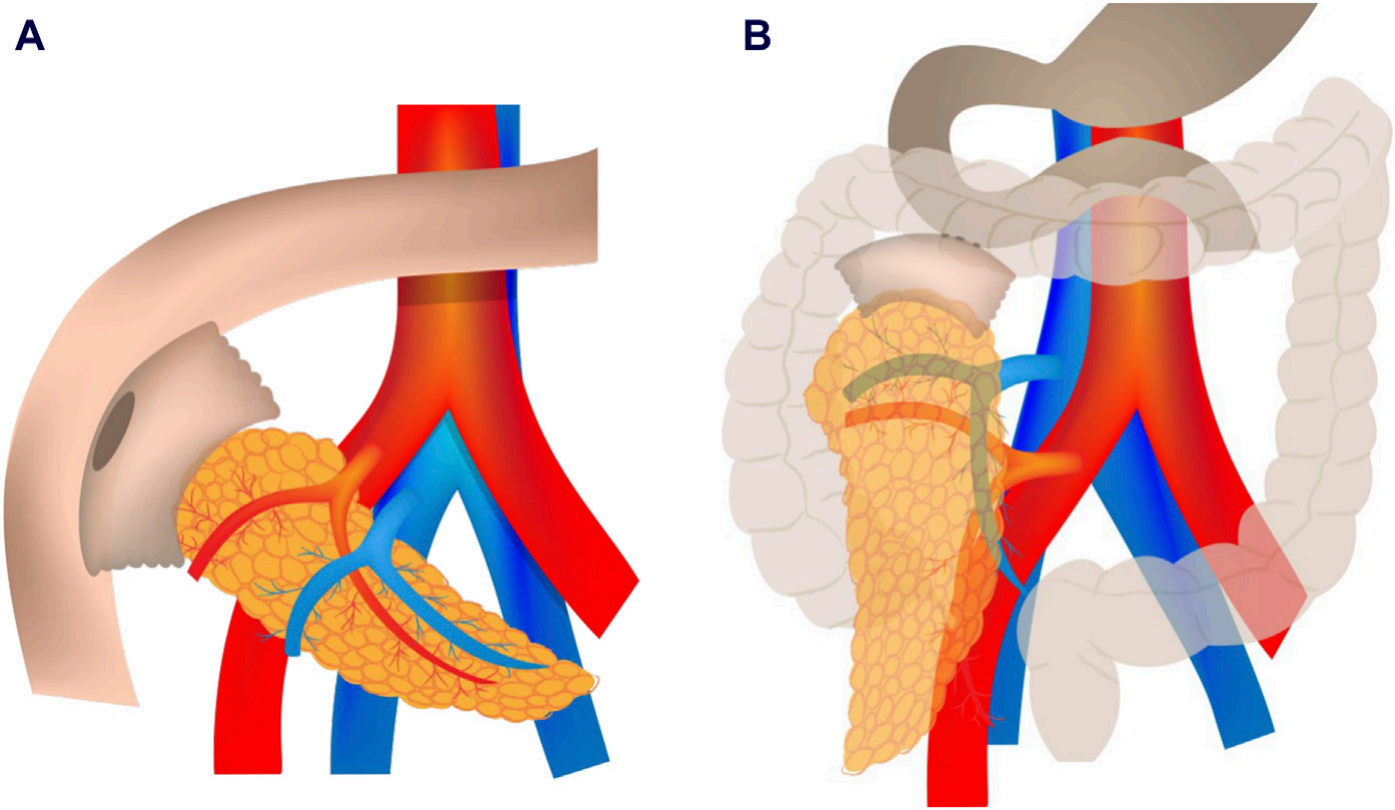
Enteric anastomosis in pancreas transplantation: classic duodeno-jejunostomy **(A)** with intraperitoneal placement and novel duodeno-duodenostomy **(B)** with retrocolic placement.

Classically, pancreatic exocrine secretions were derived to the jejunum through a duodeno-jejunostomy (DJ), placing the graft in an intraperitoneal position (Figure 1A). Recently, an alternative technique has emerged, in which the graft is placed retrocolically through a duodeno-duodenostomy (DD, Figure 1B), to mimic the physiology of the exocrine secretion in the native pancreas. In addition, it can improve the feasibility to reach the anastomotic sites [20, 21] and access to endoscopic procedures [22]. Despite the potential physiological and surgical benefits of this technique, some authors have pointed out that the retrocolic position of the pancreatic graft could limit the accessibility of percutaneous biopsy [20, 21]. Although accessibility for percutaneous graft biopsy after this surgical technique is an interesting topic, the recommendations of the first World Consensus Conference on pancreas transplantation published in 2021 [23], point out that percutaneous biopsy accessibility to retroperitoneally placed grafts still remains to be proven.

DJ intraperitoneal graft has been demonstrated to be accessible to percutaneous biopsy. To date, there are no reports on the accessibility to percutaneous biopsy on DD retrocolic grafts.

The aim of this study was to evaluate graft accessibility, the success rate for histological diagnosis and the safety of percutaneous biopsy of pancreatic grafts placed using the DD technique for enteric drainage. Furthermore, we compared these results with those obtained previously in intraperitoneal DJ grafts.

## Materials and Methods

### Study Population

The study was approved by the Institutional Ethics Committee for Clinical Research of our hospital (Reg. HCB/2020/0369). Informed consent was waived due to the retrospective nature of the study.

We conducted a retrospective study including all pancreatic grafts referred for percutaneous biopsy in our center, from the beginning of the implementation of this technique in November 2009 until July 2021. Biopsies were requested for 1) graft dysfunction (increase in serum amylase and/or lipase tripling normal value, hyperglycemia, or presence of *de novo* donor-specific antibodies) and 2) follow up of a previously treated rejection episode (4 weeks after the treatment). Furthermore, since November 2016, surveillance biopsies were requested in all patients at 3 weeks and 12 months after transplantation.

The intraperitoneal DJ with head-up graft was the stablished surgical technique employed in all pancreatic transplantations until May 2016. From this date, it was replaced by the DD, performed side-to-side by means of a hand-sewn, double layered anastomosis, returning the colon to its original position, thus completely covering the pancreas. In both cases, the venous anastomosis was performed end-to-side between donor portal vein and recipient vena cava or right iliac vein. The arterial anastomosis was constructed end-to-side between the graft superior mesenteric artery or the common iliac graft artery (depending on the backtable reconstruction as described before) [24].

### Biopsy Technique

Informed consent was obtained from all patients. All patients were required to undergo coagulation blood tests with a prothrombin time value >50% and a platelet count >50.000/mm.

All biopsies were performed by three senior radiologists with more than 10 years of experience in US- and CT-guided percutaneous biopsies. An Acuson S3000 Helx (Siemens^®^) was used with convex multifrequency (1–4.5 MHz) or linear multifrequency (4–9 MHz) transducers, depending on the depth of the graft.

Before the biopsy, a complete US B-mode study of the graft and Doppler assessment of vascular patency were conducted. Contrast-enhanced US was performed to confirm vascular permeability in cases with weak Doppler signals. After excluding vascular complications or other findings justifying graft dysfunction, the biopsy was performed.

Free-hand US guidance was the technique of choice. The percutaneous approach point was chosen based on the site with the greatest pancreatic parenchyma thickness without interposed intestinal loops, always avoiding large pancreatic vessels and the pancreatic duct if visible. The anterior approach was preferred when the patient was in a supine position, if possible. When the interposition of intestinal loops prevented the anterior approach, compression with the transducer was intended to gain access; when this maneuver did not work, a lateral approach with the patient in a decubitus lateral position or a posterior approach with the patient in the prone position was intended. If suboptimal visibility persisted after these maneuvers, a CT was performed to determine the best entry point for a posterior US-guided biopsy. If this approach was not possible, an entirely CT-guided biopsy was attempted.

The biopsy was performed after the instillation of local anesthesia (2% mepivacaine), using an automatic 18-gauge needle with a 13 mm sample length. A second sample was obtained if the first attempt yielded a sample <10 mm, with no more than three attempts.

After the procedure, firm pressure was applied at the approach point for 10 min. Patients remained admitted to the hospital 24 h after biopsy to monitor their vital signs, hematocrit, amylase and lipase levels every 4–6 h. In the absence of complications, patients were discharged within 24 h after biopsy.

Fresh graft biopsy samples were immediately sent to the pathology department for tissue processing. After formalin fixation, tissue processing and paraffin embedding of the graft biopsy, hematoxylin and eosin stains were performed for pathological analysis, and histochemical staining with Masson’s trichrome was performed for the fibrous component. Immunohistochemical staining with the antibodies CD3, CD68, insulin, glucagon, C4d, Cytomegalovirus and *in situ* hybridization for Epstein Barr virus were also performed.

All biopsy samples were examined by a single senior pathologist (MC). They were considered adequate for evaluation when sufficient to establish a diagnosis according to the Banff criteria (2011 revision) [25].

### Data Collection and Analysis

Requested biopsies were classified into two groups according to the type of surgical technique (retrocolic-DD vs. intraperitoneal-DJ). Demographic patient data, donor’s age, post-transplantation days (graft’s age), surgical technique and indication for biopsy were recorded in both groups. Data related to the biopsy procedure were also recorded for both groups: accessibility to the graft (yes/no), cause of non-accessibility, number of obtained samples, patient position when performing the biopsy, imaging guiding technique, sample adequacy for histopathological evaluation (success rate) and post-procedural complications.

The accessibility rate was calculated in both groups according to the number of performed biopsies among the total number of requested biopsies. To avoid the influence of the operator learning curve, a second analysis of the accessibility rate was performed excluding biopsies performed during the first year after the introduction of the biopsy technique (November 2009–December 2010).

As is well known, some grafts experience atrophy of the gland over time [7, 26, 27]. Thus, an analysis of the accessibility rate related to graft age was performed. To do this, all procedures were classified into five groups according to the time after transplantation: 0–3 months, 3–12 months, 1–5 years, 5–10 years and >10 years, performing a descriptive analysis of accessibility rates in each group. To avoid the influence of graft age on accessibility rate, a second subanalysis was performed that included only pancreatic grafts younger than 5 years.

### Statistical Analysis

Quantitative variables are expressed as median and interquartile range**.** Categorical variables are presented as absolute frequencies and percentages. A chi-squared test was used to compare categorical variables and the T Student test was used to compare quantitative variables. The significance level was set at 5% (two-sided).

## Results

We received a total of 268 biopsy requests in 145 patients (83 in the DJ group, 60 in the DD group and two patients with a first DJ graft and a retransplantation with a DD graft). Patient characteristics are summarized in Table 1 and data related to the biopsies are summarized in Table 2.

**TABLE 1:** Main characteristics of the two groups of patients.

	Total	DJ	DD	*p*-value
Requested biopsies (n, %)	268	136 (50.7%)	132 (49.3%)	N/A
Sex (% male)	58.6%	63.24%	53.8%	0.1165
Recipient age (median [IQR], years)	43 [37–51]	44 [38–52]	41 [36–50]	0.0046
Donor’s age (median [IQR], years)	37 [22–44]	33 [21–42]	37 [24–45]	0.0505
Post-transplant time (median [IQR], years)	10 [1–22]	20 [4–81]	3 [1–12]	<0.0001
Transplant type Simultaneous pancreas-kidney Pancreas after kidney Pancreas transplant alone Pancreas retransplant	210 53 1 4	99 35 1 1	111 18 0 3	N/A
Biopsy indication: Graft dysfunction Follow up (after rejection) Surveillance (3 weeks and 12 months)	145 (54.1%) 47 (17.5%) 76 (28.4%)	119 (87.5%) 15 (11%) 2 (1.5%)	26 (19.7%) 32 (24.2%) 74 (56.1%)	<0.0001

DJ, duodeno-jejunostomy; DD, duodeno-duondenostomy; N/A, not applicable.

**TABLE 2:** Details of the biopsy procedures.

	Total	DJ	DD	*p*-value
Requested biopsies, n	268	136	132	N/A
Performed biopsies (accessibility), n (%)	209 (78%)	93 (68.4%)	116 (87.9%)	0.0001
Guidance technique in performed biopsies (n) US CT US+CT	195 (93.3%) 8 (3.8%) 6 (2.9%)	91 (97.8%) 1 (1.1%) 1 (1.1%)	104 (89.7%) 7 (6%) 5 (4.3%)	N/A
Patient position, n (%): Supine position Lateral position Prone position	172 (82.3%) 13 (6.2%) 24 (11.5%)	92 (98.9%) 0 1 (1.1%)	80 (69%) 13 (11.2%) 23 (19.8%)	N/A
Graft not accessible, n (%) Intestinal loops interposition Graft atrophy Graft hypervascularization Liquid interposition	59 (22%) 39 11 5 4	43 (31.6%) 29 9 3 2	16 (12.1%) 10 2 2 2	0.0001
Needle passes (n) 1 2 3 Mean	166 40 3 1.22	54 36 3 1.45	112 4 0 1.03	<0.0001
Success rate for histological diagnosis, n (%)^a^	188 (89.9%)	86 (92.5%)	102 (87.9%)	0.2777
Complication rate, n (%)^a^	1 (0.5%)	0 (0%)	1 (0.8%)	N/A

DJ, duodeno-jejunostomy; DD, duodeno-duodenostomy; US, ultrasonography; CT, computed tomography; N/A, not applicable.

^a^ Success rate and complication rate are calculated over the number of performed biopsies (not the requested biopsies).

### Accessibility of the Pancreatic Graft

**FIGURE 2:**
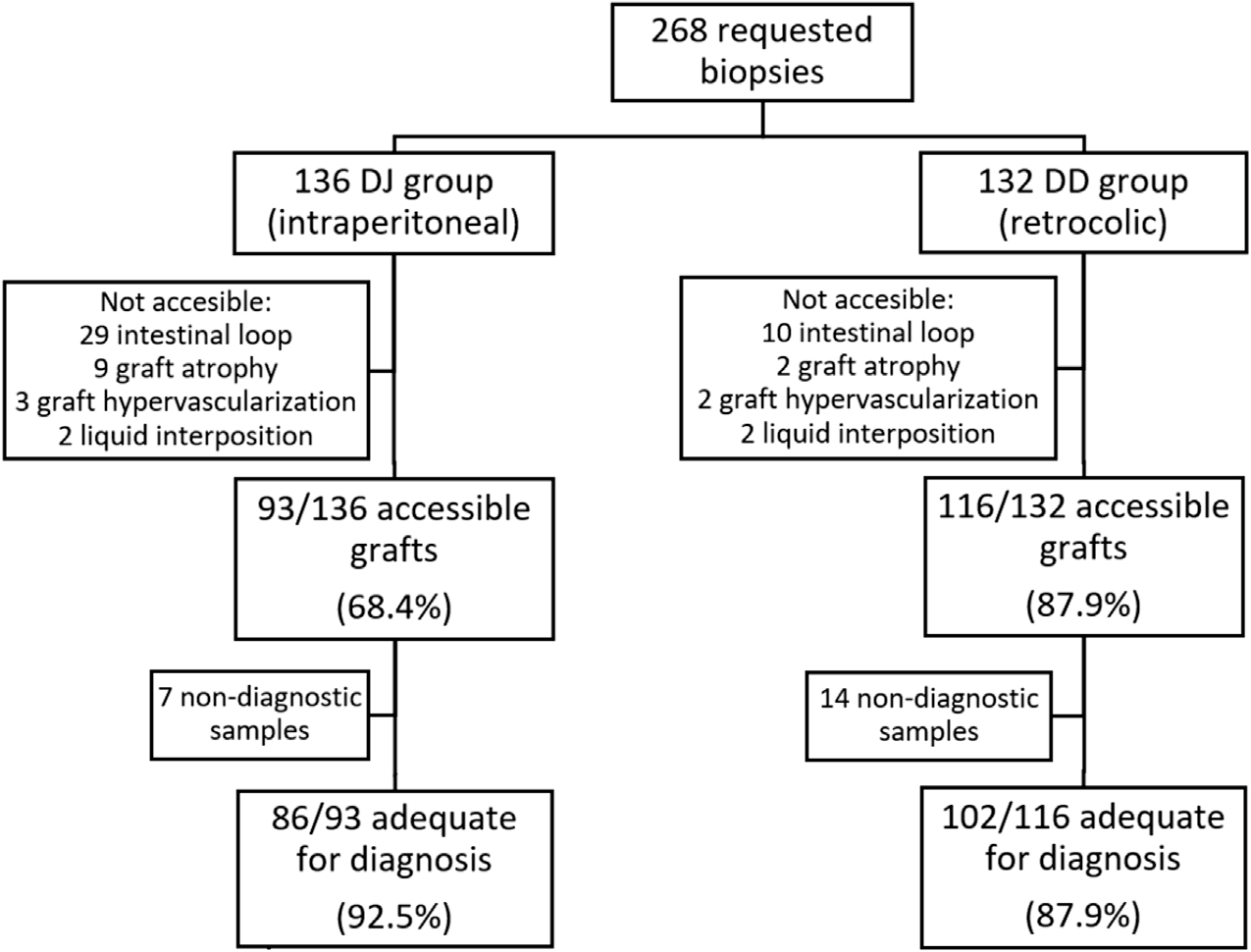
Flow-chart showing requested biopsies according to DJ and DD groups, accessibility rate and success rate for histopathological diagnosis in both groups. DJ, duodeno-jejunostomy; DD, duodeno-duodenostomy.

**FIGURE 3:**
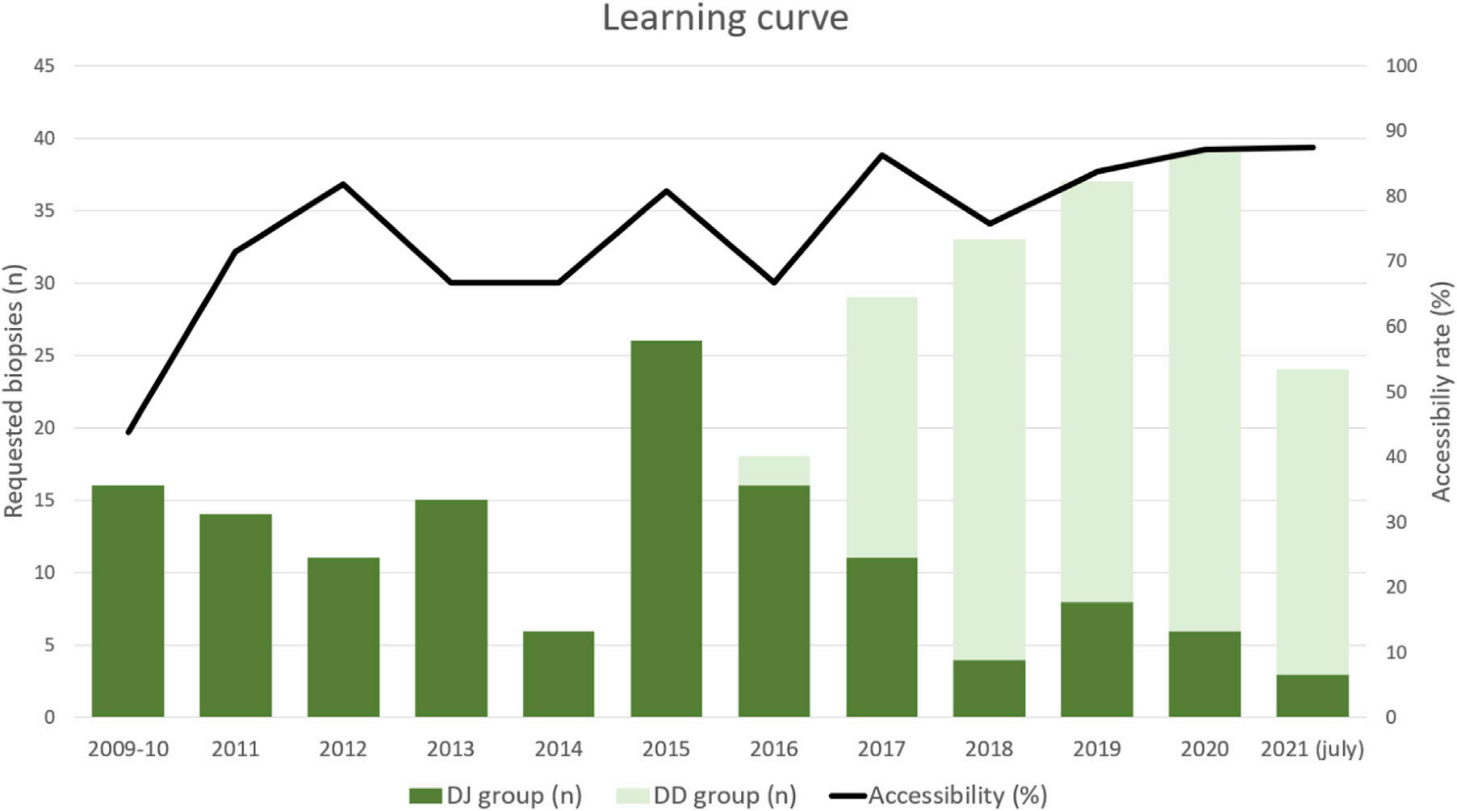
Learning curve: histogram showing the annual number of requested biopsies along the study and the total annual accessibility rate over time. DJ, duodeno-jejunostomy; DD, duodeno-duodenostomy.

As shown in Figure 2 and Table 2, the graft was accessible to biopsy in 209 out of the 268 requested biopsies (78%): 93/136 (68.4%) in DJ and 116/132 (87.9%) in DD (*p* = 0.0001). When analyzing accessibility over time (Figure 3), a lower accessibility rate was detected in the first year after implementing the biopsy procedure (43.8%). The posterior subanalysis excluding the first year (to avoid the effect of the learning curve), showed accessibility of 86/120 (71.6%) in the DJ group and 116/132 (87.9%) in the DD group, maintaining the statistical differences (*p* = 0.0022).

**FIGURE 4:**
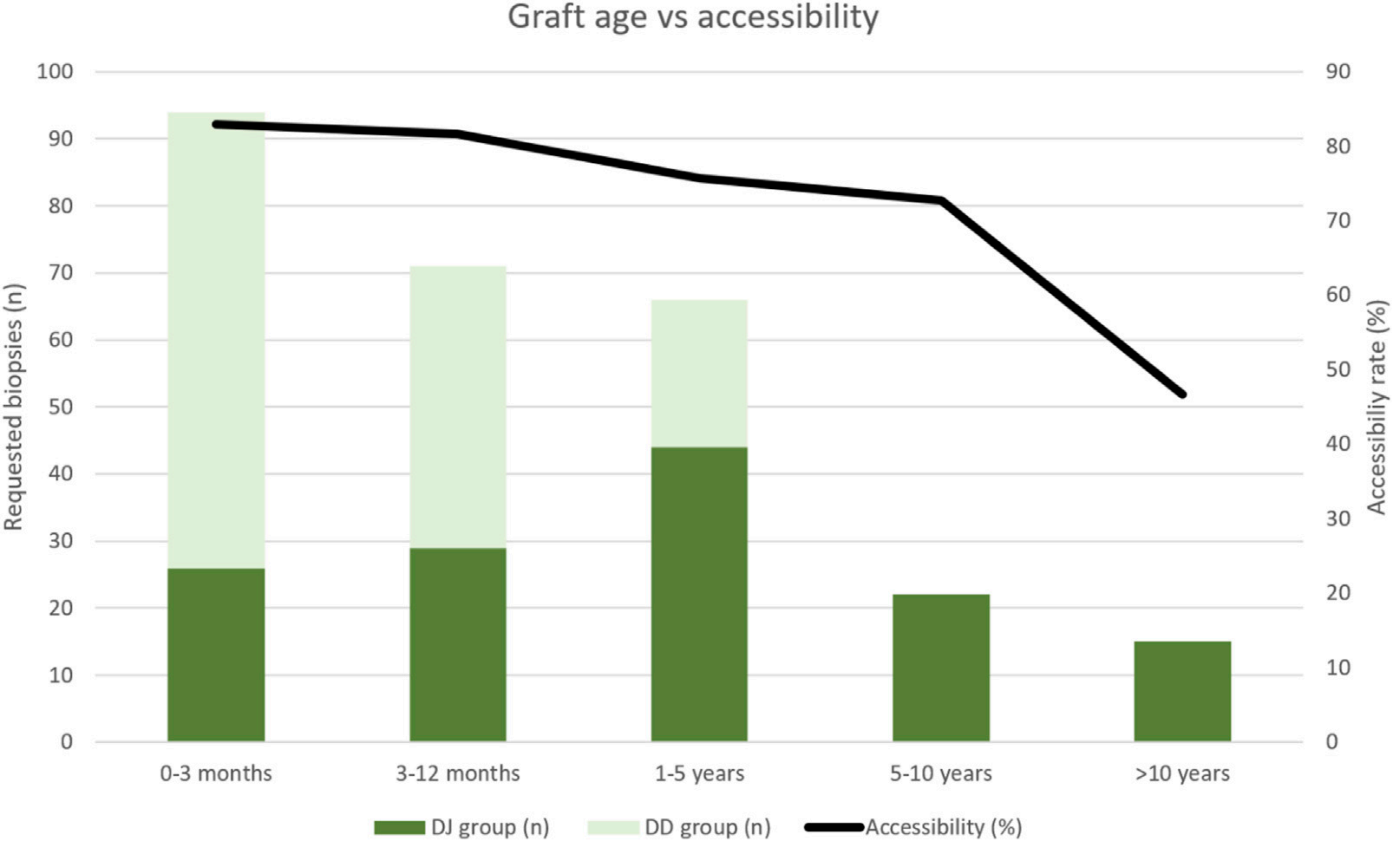
Histogram showing percutaneous biopsy accessibility rate related with the age of the graft in both groups of patients. Graft age accounts for the period of time after transplantation (in months). DJ, duodeno-jejunostomy; DD, duodeno-duodenostomy.

As shown in Table 3 and Figure 4, the highest accessibility rate was obtained when biopsy was performed during the first year after transplantation (82.4%). Subsequently, accessibility progressively decreased to 72.7% in 5–10-year-old grafts, with a significant posterior drop in grafts older than 10 years (46.7%). The additional subanalysis of accessibility including only grafts younger than 5 years (to avoid graft age bias), also showed statistical differences between the groups (70/99 in DJ and 116/132 in DD).

**TABLE 3:** Accessibility rate related to graft age and causes of not performing the biopsy in each group.

	0–3 months	3–12 months	1–5 years	5–10 years	>10 years
Requested biopsies, n (DJ:DD)	94 (26:68)	71 (29:42)	66 (44:22)	22 (22:0)	15 (15:0)
Accessibility rate (%)	83%	81.7%	75.8%	72.7%	46.7%
Graft not accessible, n (DJ:DD): Intestinal loops interposition Graft atrophy Graft hypervascularization Liquid interposition	11 (5:6) 0 3 (1:2) 2 (1:1)	8 (5:3) 1 (0:1) 2 (2:0) 2 (1:1)	12 (11:1) 4 (3:1) 0 0	1 (1:0) 5 (5:0) 0 0	7 (7:0) 1 (1:0) 0 0

DJ, duodeno-jejunostomy; DD, duodeno-duodenostomy.

US was used to guide almost all biopsies (201/209, 96.1%), six of them with the additional support of CT. The eight remaining biopsies were performed only under CT guidance due to a lack of US visibility.

The interposition of intestinal loops prevented biopsy in 76 cases (30/136 in DJ and 46/132 in DD) when the graft was assessed in the supine position. However, in 1/30 patients in the DJ group and 36/46 patients in the DD group, the graft could be accessed via a lateral or posterior approach biopsy with the patient in a lateral or prone position (Table 2). Finally, the rate of non-accessible grafts due to intestinal loop interposition was 29/136 (21.3%) in the DJ group and 10/132 (7.6%) in the DD group (*p* = 0.0001). Graft atrophy, graft hypervascularization and liquid interposition were less frequent causes of failed biopsy attempt (Table 2).

The average number of needle passes required to obtain a good sample was low (1.22), which was significantly higher in the DJ group than in the DD group as shown in Table 2.

### Success Rate for Histopathological Diagnosis

When calculated over the number of performed biopsies, in 87.9% of the DD cases, the obtained pancreatic sample was adequate to establish a histopathological diagnosis (63.2% of the requested biopsies), without statistically significant differences with the 92.5% of success rate in the DJ group (77.3% of the requested biopsies).

### Complication Rate

Only one minor complication was recorded in the DD group: an immediately mild self-limited intraabdominal hemorrhage detected by US, which did not require surgical intervention or blood transfusion (1 needle pass biopsy).

## Discussion

This study demonstrates that retrocolic pancreatic grafts placed using the DD technique are accessible to percutaneous biopsy with an accessibility rate higher than 85%. The success rate for histological diagnosis was 87.9%, which is similar to that reported for percutaneous biopsies in grafts placed with the classical surgical techniques [17, 18, 27–29]. Therefore, accessibility for a subsequent biopsy should not be a limitation to implementing the novel DD technique.

Until 2016, the intestinal drainage in our center was performed with DJ, performed side-to-side to the jejunum, 70–80 cm from the ligament of Treitz, with good results: the incidence of intestinal complications of this DJ technique from 2000 to 2016 (337 pancreas transplants) published for our group was 6.8% [30]. From this date, the DD technique was adopted successfully and with a good level of acceptance by all members of the pancreatic transplant group, with a low rate of complications (initial data published in 2017), with no intestinal complications recorded in the first 10 pancreatic DD grafts [20]. The rationale of using retrocolic graft placement over the intraperitoneal position is the easy access and dissection of the vascular anastomosis site, and easy reconstruction of venous and arterial anastomosis. To be more specific describing the surgical postoperative complications, we published in 2022, the first retrospective single-center study comparing the effects of the four most commonly used preservation solutions in PTx, i.e., UW, CS, HTK, and IGL-1, on early pancreatic graft function as well as long-term patient and graft survival. A total of 43 out of 380 cases were performed using the duodenoduodenostomy, but this fact does not affect immediate reperfusion injury rates, as vascular anastomoses were performed with the same technique throughout the time period in question. When analyzing the surgical complications according Clavien-Dindo grade no statistical differences were found between the DJ and DD groups [31]. Recently, a descriptive review of 407 pancreas transplants performed at our center (1999–2019) by analyzing the type of arterial reconstruction technique and long-term survival were published. The DD was used in 57 patients with three of them presenting with acute arterial thrombosis [24]. Due to these good results, the DD technique is the one used in our center. Initially, it was feared that this technique would limit the percutaneous biopsy accessibility, but the results of the present study demonstrate that grafts placed with the DD technique are accessible for percutaneous biopsy. In fact, in our study, accessibility was even better in the retrocolic-DD group than in the intraperitoneal-DJ group. One of the factors favoring this higher accessibility is the more cranial and posterior position of the DD graft (Figure 1B). This position offers the possibility of performing a lateral or a posterior approach, avoiding the interposition of intestinal loops, which is the main cause of not accessing the graft, both in our study and in previously reported studies, including other surgical techniques [17, 27, 28]. The lower position of DJ grafts limits the posterior and lateral approaches because the iliac bone surrounds the posterior aspect of the graft (Figure 1A). Up to 36/132 patients (27.3%) in the DD group benefited from the lateral or posterior approach (in prone or lateral patient position), thereby increasing graft accessibility from 60.6% to 87.9% in this group.

US has proven to be an excellent technique to guide percutaneous biopsy for DJ grafts [17, 18, 27, 28, 32], and it has some advantages over CT [19], as it is a faster procedure without radiation exposure. Our results demonstrated that US is also an excellent tool for guiding biopsies of retrocolic-DD graft. In our series, 89.7% of the performed biopsies in the DD group were guided by US (Table 2). This differs from the native pancreas, which is also retroperitoneal but is located in the midline position and is not accessible using the posterior approach.

In addition, percutaneous biopsy is a safe technique. Only one minor complication was recorded in the DD group, with a total complication rate of 0.5%, lower than that reported in the literature (2%–3.6%) [17, 18, 27, 28, 32]. One factor that could contribute is the low number of passes performed related to other studies [19, 27, 32]. Aideyan et al. [19], point out that CT-guided biopsy is associated with a higher risk of severe hemorrhage. This could be explained by the static imaging provided by CT, which could lead to the possibility of traversing both sides of the graft with the needle [19]. US-guided biopsy allows continuous control over the needle trajectory, which might favor a lower complication rate. The experience level of the operator could also play a role in reducing complication rates, as all our biopsies were performed by senior radiologists with vast experience in percutaneous biopsies. Another contributing factor could be the needle gauge. In our study, an 18G automatic needle was used in all patients, but no clear relationship between needle gauge and bleeding in pancreatic biopsies has been demonstrated. Lee et al. [32] compared the performances between 18G and 20G needles, reporting only a minor complication (bleeding) in the 20G group. The safety of this technique, as shown in our study, could favor the recommendation of a standardized surveillance biopsy program to detect subclinical rejection for an early treatment.

This study has some limitations. First, the DD technique was implemented in May 2016, thus all biopsies performed in the first part of the inclusion period (November 2009–May 2016) belonged to the DJ group. This means that the learning curve limitations affect only DJ patients. However, their impact on the results was reduced by excluding the biopsies performed during the first year (n = 16), which still showed significant differences in accessibility rates between groups.

Second, some grafts experience atrophy of the gland over time [7, 26, 27], potentially due to multiple episodes of undiagnosed acute rejection that may lead to chronic rejection [8, 33–36], limiting access to percutaneous biopsy. This fact is also supported by our study in which atrophy was a relevant cause for not accessing grafts older than 5 years (Table 3). Due to the implementation of the DD technique in May 2016, the DD group included patients with grafts younger than 5 years (the study ended in July 2021). Although this fact could have contributed to a decrease in the accessibility of the DJ grafts, statistically significant differences remained between groups when analyzing the accessibility rates only for young grafts (<5 years).

Third, the retrospective and monocentric nature of the study, may also be considered a limitation. But, in the scenario of pancreas transplantation with a wide variety of surgical techniques used throughout the world and the fact that it is a minority type of solid organ transplantation, it makes it difficult to carry out a multicenter study. This fact becomes more important if we take into account that the application of pancreatic biopsy in the different centers is in its infancy, both due to the worry of complications and the obvious learning curve that is needed in the context of a minority transplant. To our knowledge, this is the largest series analyzed using two different positions of the pancreatic graft, including a significant number of biopsies performed, not only in clinically indicated cases but also in those performed per protocol; without losing sight of the fact that analysis has also been carried out from an intention to treat point of view. In the absence of a reliable and proven method for the diagnosis of rejection, beyond surrogate blood analytical data, the results of the present study are of vital importance for the scientific community since it offers the possibility of making a very precise histopathological diagnosis to treat subclinical rejection with impact on long-term graft survival.

In conclusion, our results demonstrate that US-guided percutaneous biopsy of retrocolic pancreatic grafts placed by DD is a safe and effective method for the histologic diagnosis of rejection, with an accessibility rate even better than that obtained for intraperitoneal pancreatic grafts. We firmly believe that this is the first step to eliminate fears of associated morbidity to the detriment of the benefits provided, and move towards the worldwide implementation of pancreas graft percutaneous biopsy in real life to improve the outcomes of such a challenging type of transplant.

## Data Availability

The raw data supporting the conclusions of this article will be made available by the authors, without undue reservation.

## Abbreviations

CT, computed tomography; DD, duodeno-duodenostomy; DJ, duodeno-jejunostomy; US, ultrasound.
